# Time–Frequency Respiratory Impedance Maps Enable Within-Breath Deep Learning for Small Airway Dysfunction Identification

**DOI:** 10.3390/bioengineering13030280

**Published:** 2026-02-27

**Authors:** Dongfang Zhao, Sunxiaohe Li, Peng Wang, Pang Wu, Zhenfeng Li, Lidong Du, Xianxiang Chen, Ting Yang, Jingen Xia, Zhen Fang

**Affiliations:** 1 Aerospace Information Research Institute, Chinese Academy of Sciences (AIRCAS), Beijing 100190, China; zhaodongfang21@mails.ucas.ac.cn (D.Z.); linsunxiaohe23@mails.ucas.ac.cn (S.L.); wangpeng@aircas.ac.cn (P.W.); wupang@aircas.ac.cn (P.W.); lizhenfeng@aircas.ac.cn (Z.L.); lddu@mail.ie.ac.cn (L.D.); chenxx@aircas.ac.cn (X.C.); 2 School of Electronic, Electrical and Communication Engineering, University of Chinese Academy of Sciences, Beijing 100190, China; 3 Department of Pulmonary and Critical Care Medicine, Center of Respiratory Medicine, China-Japan Friendship Hospital, Beijing 100029, China; zryyyangting@163.com

**Keywords:** DeepLearning, small airway dysfunction, Impulse Oscillometry

## Abstract

Small airway dysfunction (SAD) is an early functional abnormality associated with multiple chronic airway diseases. However, clinical assessment often relies on spirometry-based indices, which require forced maneuvers and are sensitive to subject effort, thereby increasing patient burden and complicating quality control. In contrast, Impulse Oscillometry (IOS) requires only tidal breathing, imposing minimal subject burden while providing respiratory impedance indices informative for SAD identification. This study proposes a dual-domain complementary deep learning framework based on IOS for SAD identification, leveraging within-breath impedance dynamics. Specifically, raw IOS time-series signals are transformed into time–frequency respiratory impedance maps (TFRIM) capturing impedance over frequency and within-breath time. A two-stream architecture is then used to jointly learn complementary features from TFRIM and the original time-series signals. To mitigate inter-subject baseline variability, we further introduce a demographics-driven adaptive feature modulation module for subject-specific calibration. The model jointly predicts multiple small-airway indices, with decision-level fusion applied during inference. Experimental validation on 2510 subjects using five-fold cross-validation demonstrates that the proposed framework achieves an accuracy of 81.39%, outperforming representative baselines. These results suggest the potential utility of combining within-breath IOS dynamics with subject-specific calibration for SAD identification, warranting further external validation before screening deployment.

## 1. Introduction

Small Airway Dysfunction (SAD) refers to structural and functional abnormalities occurring in distal airways with an internal diameter of less than 2 mm [1]. It is widely considered an early pathological feature of several chronic airway diseases [1,2]. SAD remains prevalent in the general population, with risk linked to persistent factors such as smoking, air pollution, and demographic aging [2]. Furthermore, SAD is correlated with the presence and severity of chronic airway conditions, for example asthma. Lack of timely identification and management may contribute to poorer disease control and increased healthcare utilization, thereby adding to the overall clinical and economic burden [3]. Therefore, accurate and timely identification of SAD is clinically important.

In clinical practice, spirometric indices are commonly used to assess small airway function. This method requires subjects to perform a maximal forced expiration following a deep inspiration. Such reliance on maximal subject effort places substantial demands on patient cooperation and physical capacity. Moreover, significant variability may stem from differences in effort and technique, thereby complicating quality assurance [4]. These limitations may hinder the routine detection of SAD, particularly in individuals who cannot reliably perform forced maneuvers and in settings with limited resources. Therefore, there is a distinct clinical need for a detection technique that is independent of forced maneuvers to support broader screening.

To address this challenge, researchers have explored various methods for SAD identification that do not rely on forced maneuvers. Among these, the Multiple Breath Nitrogen Washout (MBNW) test is regarded as sensitive for detecting ventilation inhomogeneity [5,6]. However, its widespread adoption is limited by high equipment costs and lengthy testing protocols. Body plethysmography can provide global respiratory mechanics data, such as lung volume and airway resistance, but it is less sensitive to small airway dysfunction [7]. Some studies have also applied High Resolution Computed Tomography (HRCT) for anatomical assessment. Nevertheless, HRCT is limited by insufficient spatial resolution to visualize the small airways and exposes patients to ionizing radiation, making it unsuitable for mass screening [8,9]. More broadly, multimodal phenotyping strategies that integrate functional testing with biological and imaging markers are increasingly advocated to better characterize airway pathology and its systemic implications [10]. In particular, cardiopulmonary imaging-derived phenotypes enable structure–function correlation analyses that refine airway disease subtypes and improve interpretability [11]. However, such multimodal and imaging-centric workflows may remain resource-intensive for large-scale screening. Consequently, there remains a need for a low-cost, noninvasive approach that can be widely deployed for screening and is sensitive to small airway dysfunction. Impulse Oscillometry (IOS) is a noninvasive lung function test performed during tidal breathing. It quantifies respiratory system impedance across a range of frequencies. Low-frequency indices have been reported to reflect mechanical changes in the peripheral airways, making them valuable as adjunct measures for SAD assessment [12,13]. While prior studies have explored the use of IOS for SAD identification, existing methodologies commonly focus on averaged scalar parameters [14,15]. However, relying solely on averaged scalars amounts to a dimensionality reduction of the raw time-series data. Such breath-averaged summaries may be insufficient to capture time-varying respiratory impedance within each tidal breath, potentially overlooking phase-dependent and nonlinear impedance behaviors during tidal breathing. Furthermore, these approaches may not fully account for the influence of demographic factors on baseline respiratory mechanics [16]. Without subject-specific normalization, this variability across subjects can confound disease-related patterns and impair generalization across cohorts. Consequently, leveraging within-breath dynamics from raw IOS signals, while incorporating subject-specific calibration to address baseline variability, offers a strategy to improve both the accuracy and robustness of SAD identification.

Concurrently, recent years have witnessed rapid progress in machine learning for biomedical data analysis, ranging from physiological time-series modeling to medical image interpretation. These advances enable automated extraction of clinically meaningful patterns from complex and noisy measurements [17,18,19,20,21,22,23]. In respiratory medicine, computational phenotyping is evolving to characterize airway pathologies across heterogeneous phenotypes, while advanced signal-processing representations and deep learning further facilitate modeling of non-stationary, within-breath respiratory dynamics beyond simple breath-averaged scalars. Specifically for oscillometry, modern sequence-learning architectures, together with principled signal representations, offer effective tools to model phase-dependent variations in respiratory mechanics, thereby supporting advanced IOS-based SAD analysis.

Motivated by clinical need and leveraging the aforementioned computational advancements, we propose a dual-domain complementary deep learning identification framework that emphasizes within-breath dynamics and subject-specific feature space calibration to leverage information in raw IOS signals and enhance robustness to inter-subject variability. Within this framework, we transform raw IOS time-series into a time–frequency respiratory impedance map (TFRIM), representing impedance as a joint function of frequency and within-breath time. This design is supported by previous clinical studies, which have reported associations between SAD and variations in impedance over the respiratory cycle [24,25]. Compared with approaches based on cycle-averaged scalar parameters, TFRIM may better preserve dynamics that vary across the respiratory cycle and are often attenuated by static summaries. To jointly leverage complementary information from both TFRIM and raw time-series signals, the proposed framework adopts a two-stream architecture. Specifically, one stream operates on the TFRIM and the other on the raw time-series. Furthermore, we introduce a demographics-driven feature space calibration mechanism to mitigate baseline discrepancies across subjects and improve robustness to inter-subject variability. We formulate SAD identification as a multi-task learning problem by jointly predicting multiple small airway indices. During inference, the final decision is obtained via decision-level fusion across subtask predictions. The main contributions of this study are summarized as follows:

- Dual-Domain Framework: We develop a dual-domain deep learning framework that jointly processes TFRIM and raw time-series signals.
- We introduce TFRIM to extend traditional IOS analysis from static parameters to the dynamic time–frequency domain, enabling the representation of impedance patterns that vary across the respiratory cycle and may be relevant to SAD.
- Feature Space Calibration: We introduce a demographics-driven feature-space calibration mechanism to mitigate baseline discrepancies across subjects and improve robustness to inter-subject variability.
- Experimental Evaluation: Cross-validation on the study dataset indicates that the proposed method demonstrates improved or comparable performance relative to representative baselines, warranting further investigation for potential clinical utility.

## 2. Materials and Methods

### 2.1. Impulse Oscillometry

IOS is a noninvasive technique for assessing pulmonary function. A distinct advantage of IOS is that it requires only quiet tidal breathing from the subject, thereby eliminating the need for forced respiratory maneuvers.

The fundamental principle involves applying a weak pressure pulse, composed of multiple frequency components, to the respiratory system via the mouth during tidal breathing. Simultaneously, the resulting variations in oral airflow are measured. During the acquisition period *T*, the device synchronously records the time-domain pressure signal p[n] and flow signal f[n] at a sampling rate of $f_{s}=400\;\mathrm{Hz}$, where n=0,1,…,N−1 (with $N=T\cdot f_{s}$).

In standard clinical practice, the total respiratory impedance Z[k] is derived as the frequency-domain ratio of these signals using the Discrete Fourier Transform (DFT):

(1) $$Z\left[k\right]=\frac{P[k]}{F[k]}=R\left[k\right]+jX\left[k\right],k=0,\ldots ,N-1$$

Here, P[k] and F[k] denote the one-sided DFT spectra of the pressure and flow signals, respectively. Because p[n] and f[n] are real-valued signals, the impedance spectrum is symmetric. Therefore, in the subsequent analysis, we retain only the one-sided frequency components k=0,…,⌊N/2⌋. R[k] and X[k] represent the resistance and reactance at the *k*-th frequency, respectively. Specifically, respiratory resistance *R* primarily reflects energy dissipation caused by friction as airflow traverses the respiratory tract, serving as a critical index for assessing airway patency. Conversely, respiratory reactance *X* reflects the elastic properties of the lung and chest wall, as well as the inertia of the airflow [26]. Notably, *X* is highly sensitive to peripheral airway dysfunction and parenchymal pathology, providing physiological insights that extend beyond traditional pulmonary function testing [13].

However, Equation (1) calculates a global average impedance based on the assumption that signals remain stationary throughout the acquisition period. This methodology obscures the dynamic fluctuations in airway resistance that occur within the respiratory cycle. To address this limitation, this study moves away from relying on traditional static parameters R[k] and X[k]. Instead, we construct TFRIM directly from the raw discrete time-series sequences p[n] and f[n] to capture time-varying characteristics.

### 2.2. Model Architecture

**Figure 1:**
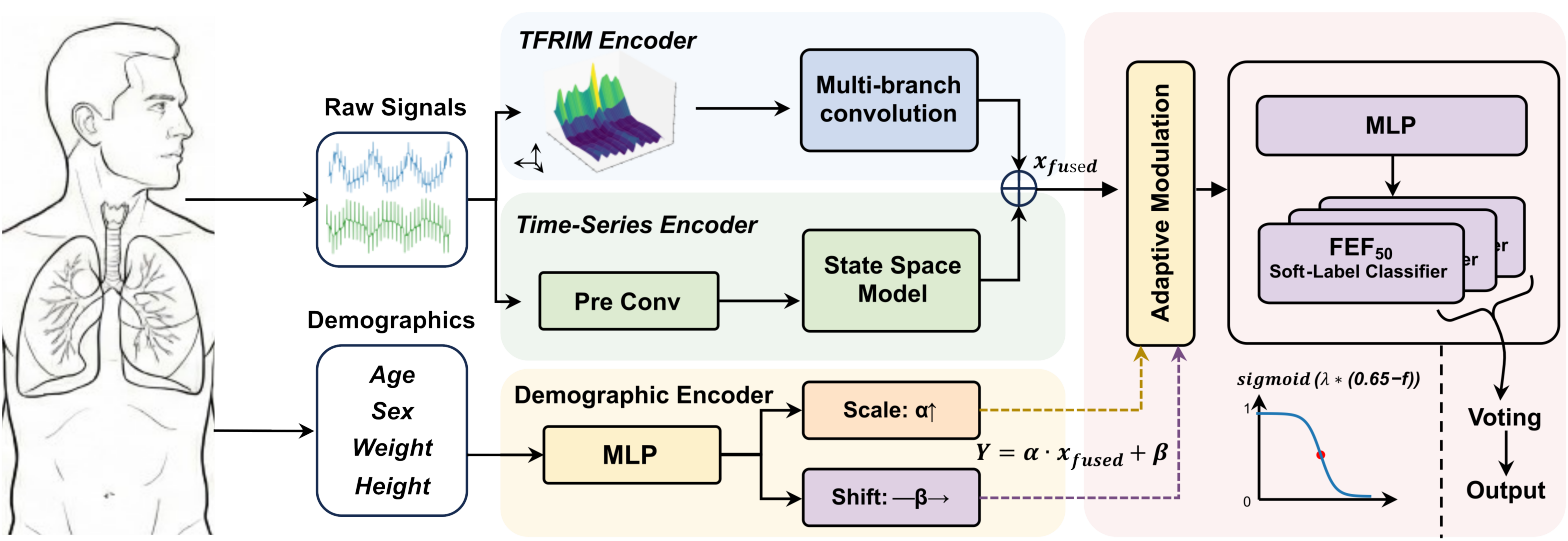
Overall architecture of the proposed dual-domain complementary deep learning framework for SAD identification.

This study proposes a dual-domain complementary deep learning identification architecture. This architecture incorporates clinical prior knowledge and aims to achieve precise identification of SAD through multi-dimensional representation learning. As illustrated in Figure 1, the overall architecture draws inspiration from the decision-making paradigm of clinicians, involving comprehensive identification based on multi-source information. It integrates the spectral texture features of TFRIM, the waveform dynamics of raw IOS time-series signals, and the demographic characteristics of the subjects. Through this integration, the model achieves a multi-dimensional assessment of airway functional status. Specifically, the data flow extracts features from different domains via two encoding paths within the network. Subsequently, these features undergo feature fusion, adaptive calibration, and decision fusion to output the final identification result.

Specifically, the front-end of the network is designed as a dual-stream architecture, comprising a TFRIM encoding branch and a time-series encoding branch. The TFRIM encoding branch adopts a convolutional neural network (CNN) architecture to capture the time–frequency impedance texture features embedded within the respiratory cycle. The time-series encoding branch employs a Mamba architecture to directly process raw one-dimensional discrete time sequences. This branch efficiently models long-range dependencies and extracts instantaneous waveform features in an end-to-end manner. These two branches provide information representations from different dimensions, collectively constituting a characterization of respiratory dynamics.

Furthermore, to alleviate the issue of inter-individual representation discrepancies caused by physiological differences, we designed a demographic feature encoding branch. This branch maps demographic features to affine transformation parameters via a Multi-Layer Perceptron (MLP). In the Demographics-driven Adaptive Feature Modulation (DAFM) module, the deep features output by the first two branches are first concatenated and fused. Subsequently, these features are dynamically calibrated using the affine transformation parameters. This process aims to mitigate the impact of physiological differences on model identification.

At the prediction stage, we draw inspiration from spirometry-based clinical assessment and adopt a soft label multi-task learning strategy. In clinical spirometry, SAD is commonly assessed using expiratory flow indices derived from the forced vital capacity (FVC) maneuver, including the mean forced expiratory flow between 25% and 75% of FVC (FEF_25–75_), the forced expiratory flow when 50% of FVC has been exhaled (FEF_50_), and that when 75% of FVC has been exhaled (FEF_75_). Accordingly, the network terminates in three parallel classification heads, each predicting whether the corresponding spirometry-derived index falls below its predefined threshold. The final SAD decision is obtained via majority voting over the three head predictions, which may reduce reliance on any single index and facilitate clinically aligned interpretation of the outputs.

#### 2.2.1. Construction of TFRIM

To capture dynamic physiological features related to SAD, we first constructed the TFRIM based on the raw IOS signals. Specifically, we performed a Short-Time Fourier Transform (STFT) on the synchronously acquired discrete pressure signal p[n] and flow signal f[n]. In this process, we employed a Hamming window with a length of 4 s and sliced the signal with a step size of 0.4 s. Considering the inevitable noise interference in respiratory signals, we did not use simple spectral division to calculate impedance for each time window *t*. Instead, we adopted the cross-power spectral density estimation method, which is more robust to noise [27]. The instantaneous impedance $Z_{t}\left[k\right]$ within the *t*-th time window is calculated as follows:

(2) $$Z_{t}\left[k\right]=\frac{S_{PF}^{\left(t\right)}\left[k\right]}{S_{FF}^{\left(t\right)}\left[k\right]}=\frac{P_{t}\left[k\right]F_{t}^{*}\left[k\right]}{F_{t}\left[k\right]F_{t}^{*}\left[k\right]}=R_{t}\left[k\right]+jX_{t}\left[k\right]$$

where $P_{t}\left[k\right]$ and $F_{t}\left[k\right]$ denote the STFT spectra of the pressure and flow signals within the current window, respectively. $P_{t}^{*}\left[k\right]$ and $F_{t}^{*}\left[k\right]$ represent their complex conjugates. $S_{PF}\left[k\right]$ is the cross-power spectral density of the pressure and airflow signals, while $S_{FF}\left[k\right]$ is the auto-power spectral density of the airflow signal. This method utilizes the autocorrelation of the flow signal. By doing so, it effectively suppresses the influence of measurement noise.

Finally, we split the impedance spectrum $Z_{t}\left[k\right]$ calculated for all time windows *t* into resistance $R_{t}\left[k\right]$ and reactance $X_{t}\left[k\right]$. Subsequently, we stacked them along the time axis to construct the raw Time–Frequency Respiratory Impedance Map (raw-TFRIM). Each sample of raw-TFRIM contains two channels, representing the variations of airway resistance and reactance over time at different frequencies.

The external excitation signal of the IOS is a periodic pulse signal with a fundamental frequency of 2.5 Hz. Its energy in the frequency domain is concentrated at the fundamental frequency and its harmonics, rather than covering the full frequency band. Therefore, the impedance values in the raw-TFRIM do not possess sufficient Signal-to-Noise Ratio (SNR) at all frequencies. To eliminate the interference of low-SNR frequency bands on the model, we designed a frequency screening strategy based on the coherence function $\gamma^{2}$.

The coherence function is used to quantify the linearity of the system and the causality between input and output. It is a common metric for evaluating the quality of impedance calculation [28]. We set the coherence threshold to $\gamma_{th}=0.90$ as a stringent quality-control criterion to exclude low-coherence frequency bins, and screened each frequency point *k* as follows:

(3) $$\begin{array}{c} \gamma_{t}^{2}\left[k\right]=\frac{|S_{PF}{\left[k\right]|}^{2}}{S_{PP}\left[k\right]\cdot S_{FF}\left[k\right]}\\ {\hat{Z}}_{t}\left[k\right]=\left\{\begin{array}{cc} Z_{t}\left[k\right], & \gamma_{t}^{2}\left[k\right]\geq \gamma_{th}\\ 0, & \gamma_{t}^{2}\left[k\right]<\gamma_{th} \end{array}\right. \end{array}$$

where $\gamma_{t}^{2}\left[k\right]$ represents the coherence function value at that frequency point, and $S_{PP}$ is the auto-power spectral density of the pressure signal. To further optimize computational efficiency, we removed the frequency channels set to zero by Equation (3) from the raw-TFRIM. Thus, we retained only the core frequency channels containing high-SNR information.

Through this frequency selection strategy based on the coherence function, we refined the TFRIM containing only high-SNR impedance values from the raw-TFRIM. This serves as the input for the model, establishing a data foundation for the subsequent model to achieve accurate and robust identification. The dimensions of the TFRIM finally input into the model are $C_{TF}\times H_{TF}\times W_{TF}$. Here, $C_{TF}=2$ represents the two channels of resistance and reactance, $H_{TF}$ represents the effective frequency channels after screening, and $W_{TF}$ represents the number of time windows.

#### 2.2.2. TFRIM Encoding Branch

**Figure 2:**
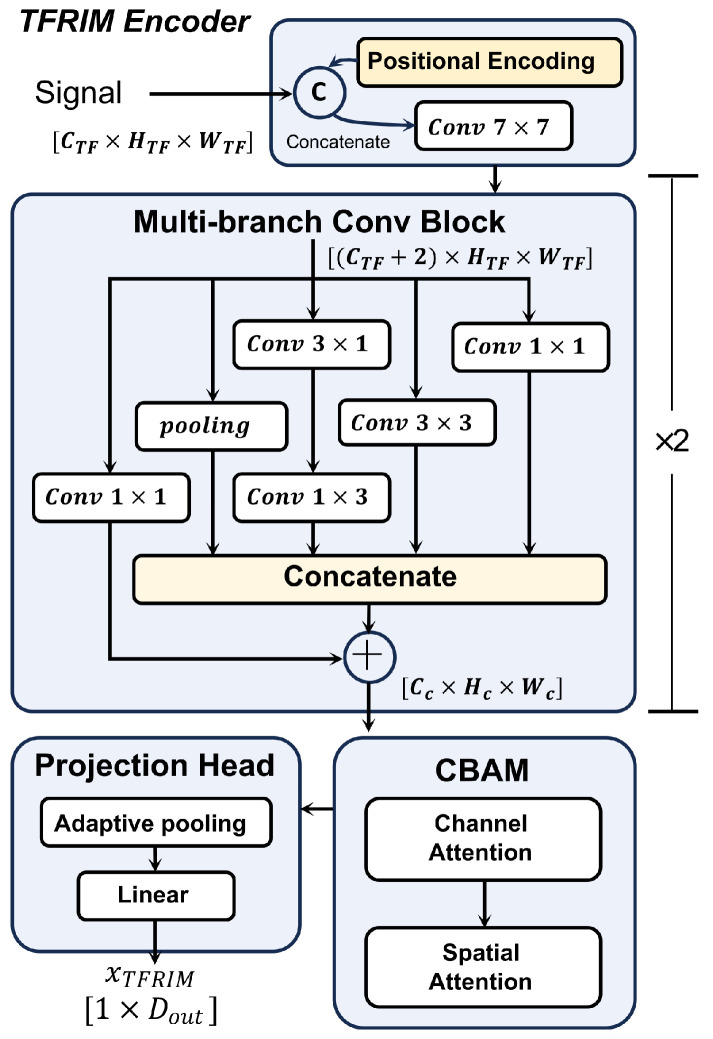
Architecture of the TFRIM encoding branch with position encoding, multi-branch convolution modules, and CBAM-based attention.

The TFRIM encoder branch is designed based on a CNN architecture, as illustrated in Figure 2. Unlike natural images, the two dimensions of TFRIM possess distinct and heterogeneous physical meanings. While the inherent translational invariance of CNNs is beneficial for object identification, in this specific scenario, it causes the model to ignore the absolute position of features in the frequency spectrum [29]. To address this issue, we explicitly constructed a two-channel position encoding map consistent with the dimensions of the TFRIM. This map encodes the normalized time and frequency coordinates, respectively. Finally, the position encodings are concatenated with the TFRIM along the channel dimension. The concatenated data is processed by a convolutional layer with a kernel size of 7 to serve as the initial input for the multi-branch convolution module.

The core of the TFRIM encoder branch consists of two cascaded multi-branch convolution modules, aimed at capturing multi-scale time–frequency features. The module employs a topology combining multi-path parallel processing with residual connections. It contains four parallel internal feature extraction branches. The first branch consists of a pooling layer, designed to capture background information in local regions. The second branch cascades a 3×1 convolutional layer and a 1×3 convolutional layer. This asymmetric design decouples the receptive field. It enables the model to independently learn impedance distribution patterns along the frequency axis and respiratory phase characteristics along the time axis. The third branch uses a standard 3×3 convolution to capture conventional local time–frequency features. The fourth branch employs a 1×1 convolution, aiming to facilitate cross-channel information interaction and fusion. This helps in extracting more expressive features and enhancing the model’s learning capability. The outputs of these four internal branches are first concatenated along the channel dimension to form a high-dimensional feature description. To promote effective gradient propagation and resolve dimensionality mismatch, we introduced an external residual connection branch. This branch uses a 1×1 convolution to map the input features to the same dimension as the concatenated features. Finally, feature aggregation is completed via element-wise addition.

The aggregated high-dimensional features are further fed into a Convolutional Block Attention Module (CBAM). This module sequentially computes attention maps in the channel and spatial domains. This enables the model to adaptively focus on channels and spatial locations with high attention [30].

Finally, the feature maps are compressed into a one-dimensional vector via global adaptive average pooling. Subsequently, they are mapped to a 128-dimensional latent feature space through a linear projection layer containing ReLU activation and Dropout regularization, facilitating subsequent feature fusion with the time-series branch. Ultimately, the time–frequency feature vector $X_{\mathrm{TFRIM}}\in R^{128}$ is obtained via this branch encoding.

#### 2.2.3. Temporal Encoding Branch

**Figure 3:**
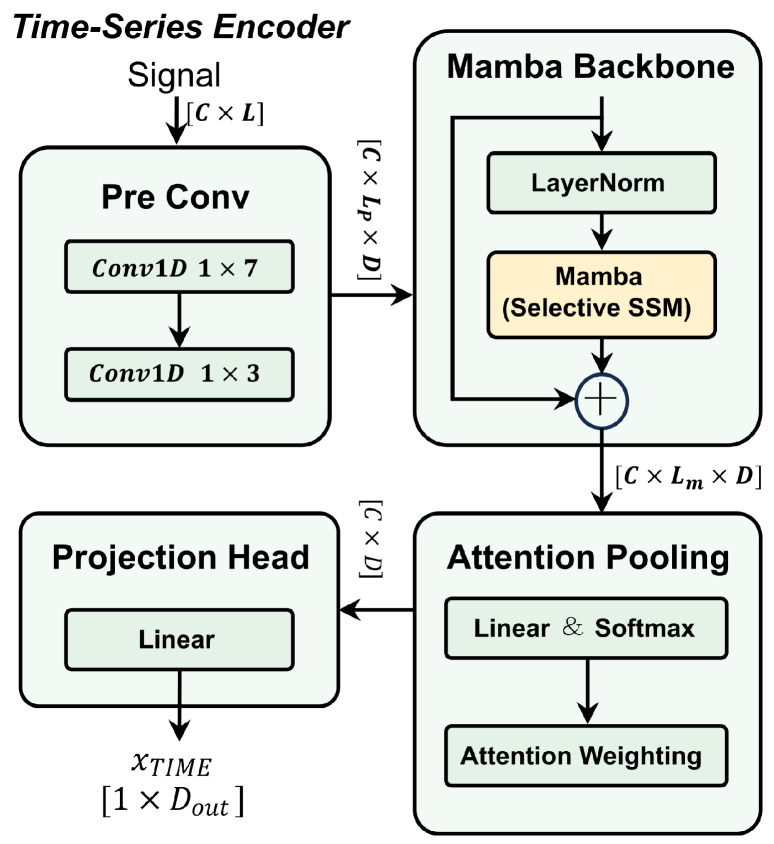
Architecture of the temporal encoding branch based on Mamba with local feature extraction and attention pooling.

To extract deep physiological features from high-sampling-rate raw IOS time-series signals, this study designs a temporal encoder branch based on Mamba. As shown in Figure 3, this branch consists of a lightweight local feature extraction module, a Mamba module, and an attention pooling layer.

The front end of the time-series encoder branch is composed of a feature extraction module. Addressing the high-sampling-rate nature of raw IOS signals and the accompanying measurement noise, this module aims to achieve signal denoising and preliminary feature extraction through a multi-layer convolutional structure. Specifically, the first convolutional layer of this module is configured with a large kernel size of 7 and a stride of 2. This configuration effectively compresses redundancy in the temporal dimension while expanding the initial receptive field. After processing by this module, the raw time-series signals are mapped into compact local feature embeddings $F_{\mathrm{local}}\in R^{C\times L_{P}\times D}$, which serve as the input for the subsequent network. Here, *C* indicates the number of input signal channels, corresponding to the simultaneously recorded pressure and flow waveforms. The symbol $L_{P}$ denotes the temporal length of the resulting feature sequence after the local convolutional extractor performs temporal downsampling. *D* corresponds to the feature embedding dimension produced by the local convolutional extractor.

Furthermore, we inject $F_{\mathrm{local}}$ into the Mamba module to capture the cross-cycle dynamic variation patterns within long-sequence respiratory signals [31]. This module is built upon the theoretical framework of the SSM. It introduces a selectivity mechanism to overcome the limitations of traditional Linear Time-Invariant (LTI) models. Formally, we treat $F_{\mathrm{local}}$ as *D* independent one-dimensional respiratory feature sequences x(t) for subsequent computation. The model views these as continuous systems and maps them to the output y(t) through the hidden state h(t). The continuous-time dynamic equations can be expressed as follows:

(4) $$h^{\prime }\left(t\right)=Ah\left(t\right)+bx\left(t\right)$$

(5) $$y\left(t\right)=ch\left(t\right)+D_{\mathrm{skip}}x\left(t\right)$$

Here, $A\in R^{N\times N}$ denotes the diagonal state evolution matrix, and $D_{\mathrm{skip}}\in R^{1}$ represents the position-independent skip connection parameter. Unlike traditional SSM, Mamba introduces an input-dependent parameterization strategy. Specifically, we define the time scale parameter Δ, as well as $b\in R^{N\times 1}$ and $c\in R^{1\times N}$, as functions of the current input $x_{t}$:

(6) $$\begin{array}{c} \Delta_{t}=\mathrm{softplus}\left(w_{\Delta }x_{t}+b_{\Delta }\right) \end{array}$$

(7) $$\begin{array}{c} b_{t}=w_{b}x_{t} \end{array}$$

(8) $$\begin{array}{c} c_{t}=w_{c}x_{t} \end{array}$$

Here, $w_{\Delta }$ and $b_{\Delta }$ denote trainable projection parameters that map the current input feature $x_{t}$ to the positive time-scale parameter $\Delta_{t}$ through a softplus nonlinearity. These parameters are learned jointly with the whole network under the same optimization objective. Similarly, $w_{b}$ and $w_{c}$ are trainable projection matrices used to generate the input-dependent state-space parameters $b_{t}$ and $c_{t}$ respectively. Given that IOS signals are discretely sampled digital signals, we employ the Zero-Order Hold (ZOH) principle to discretize the aforementioned continuous equations. The discretized parameters are calculated as follows:

(9) $$\begin{array}{c} {\bar{A}}_{t}=\exp\left(\Delta_{t}A\right) \end{array}$$

(10) $$\begin{array}{c} {\bar{b}}_{t}=\left(\exp\left(\Delta_{t}A\right)-I\right)\cdot A^{-1}b_{t} \end{array}$$

Based on this, the discretized state space model can be recursively expressed as:

(11) $$\begin{array}{c} h_{t}={\bar{A}}_{t}h_{t-1}+{\bar{b}}_{t}x_{t} \end{array}$$

(12) $$\begin{array}{c} y_{t}=c_{t}h_{t}+D_{\mathrm{skip}}x_{t} \end{array}$$

Through this parameterization mechanism, the model is capable of dynamically adjusting the time scale Δ and state transition weights according to the current respiratory signal characteristics. This capability enables the selective memory of key features within the latent space while effectively suppressing irrelevant background noise. Ultimately, the module employs a residual architecture with pre-layer normalization to output the feature sequence *Z*:

(13) $$Z=\mathrm{Mamba}\left(\mathrm{LayerNorm}\left(F_{\mathrm{local}}\right)\right)+F_{\mathrm{local}}$$

Given that the pathological manifestations of SAD are dependent on respiratory phases, traditional global average pooling strategies exhibit limitations as they tend to dilute key information with background signals from healthy phases. To address this, we deployed an attention pooling layer at the end of the network. This layer aims to automatically evaluate the value of each time step in the feature sequence via learnable weight parameters. The model first calculates the normalized attention score $\alpha_{t}$ for each time step *t* and subsequently performs a weighted aggregation of the feature sequence *Z* based on these scores:

(14) $$e_{t}=w\cdot z_{t}$$

(15) $$\alpha_{t}=\frac{\exp(e_{t})}{\sum_{\tau =0}^{T}\exp\left(e_{\tau }\right)}$$

(16) $$v_{\mathrm{context}}=\sum_{t=0}^{T}\alpha_{t}z_{t}$$

In these equations, $z_{t}\in R^{D}$ denotes the *D*-dimensional latent feature at time *t*, and $w_{t}\in R^{D}$ represents the learnable attention weight vector. Furthermore, $\alpha_{t}\in R$ is the normalized scalar attention score, while $v_{\mathrm{context}}$ serves as the context vector after adaptive focusing.

To enable the effective fusion of this time-domain feature with the features extracted by the TFRIM encoder branch, we further introduced a feature projection module. Specifically, we map $v_{\mathrm{context}}$ to a 128-dimensional high-dimensional subspace via a fully connected layer, ensuring consistency with the dimension of the TFRIM branch. Following this projection operation, a ReLU activation function and a Dropout layer are incorporated to enhance the non-linear expressive capability of the features and the generalization performance of the model. Consequently, this process generates the time-series feature vector $x_{\mathrm{TIME}}\in R^{128}$, which is utilized for multi-branch joint identification.

#### 2.2.4. Demographic Characteristics Encoding Branch

Due to demographic differences, subjects with the same pathological state may present distinct feature distributions, which can confound models. To suppress the interference of such inter-individual physiological differences on model decision-making, this study designed a demographics-based encoding branch. This branch is primarily composed of an MLP and a DAFM. The core idea is to utilize demographic characteristics to dynamically generate modulation parameters. In this way, the branch aims to reduce the influence of physiological variations among subjects.

First, in this module, we concatenate the feature vector $X_{TFRIM}\in R^{128}$ output by the TFRIM encoding branch with the feature vector $X_{TIME}\in R^{128}$ output by the time-series encoding branch. Through this process, we obtain the fused feature vector $X_{fused}$ as described in Equation (17).

(17) $$x_{\mathrm{fused}}=\mathrm{Concat}\left(x_{\mathrm{TFRIM}},x_{\mathrm{TIME}}\right)\in R^{256}$$

Simultaneously, we input the standardized demographic feature vector $d\in R^{4}$ into an MLP containing three hidden layers. This MLP is designed to learn a non-linear mapping from the low-dimensional demographic feature space to the high-dimensional modulation parameter feature space. Its output is projected to the $R^{512}$ dimension. Subsequently, it is split into two parameter vectors, both with a dimension of 256. These two vectors constitute the scaling factor α and the shifting factor β, respectively, as shown in Equation (18).

(18) [α,β]=MLP(d)

Here, $\alpha ,\beta \in R^{256}$. Finally, we utilize these two parameter vectors within the DAFM module. We perform element-wise modulation on the fused feature $X_{fused}$ to generate the calibrated feature vector $X_{mod}$, as formulated in Equation (19).

(19) $$x_{\mathrm{mod}}=\alpha ⊙x_{\mathrm{fused}}+\beta$$

In this equation, ⊙ represents element-wise multiplication. Through this mechanism, the model is capable of adaptively enhancing or suppressing the feature distribution of specific channels based on the demographic characteristics of each subject. This consequently improves the identification accuracy of the model among populations with diverse physiological features. The calibrated feature $x_{\mathrm{mod}}$ is subsequently fed into a multi-label classifier for the final identification of clinical indices.

### 2.3. Model Training

To guide the model to attend to more clinically meaningful features, we introduce the identification of clinical indices as finer-grained tasks. Accordingly, this study formulates the training objective as a multi-label classification problem. Specifically, the classification head of the model is designed as a shared-weight MLP. It receives the calibrated feature vector $x_{\mathrm{mod}}$ from the previous stage as input and maps it to a three-dimensional vector of logits z.

(20) $$z={\left[z_{1},z_{2},z_{3}\right]}^{⊤}=\mathrm{MLP}\left(x_{\mathrm{mod}}\right)\in R^{3}$$

In this study, each element in the vector z corresponds to the probability logits for three key clinical indices, FEF_25–75_, FEF_50_, and FEF_75_. Subsequently, we transform these logits into predicted probabilities $\hat{y}=\left[\sigma \left(z_{1}\right),\sigma \left(z_{2}\right),\sigma \left(z_{3}\right)\right]\in R^{3}$ within the interval (0,1) via the Sigmoid function.

We define the loss function of the model as the multi-label binary cross-entropy loss between the predicted probabilities $\hat{y}$ and the soft labels $y_{soft}^{\left(k\right)}$. The model is optimized by minimizing this loss function. For a single sample, the total loss is defined as shown in Equation (21):

(21) $$L=-\frac{1}{K}\sum_{k=1}^{K}\left[y_{soft}^{k}\cdot \log\left({\hat{y}}_{k}\right)+\left(1-y_{soft}^{k}\right)\cdot \log\left(1-{\hat{y}}_{k}\right)\right]$$

In this equation, K=3 represents the three sub-labels in this study, while $y_{k}$ and ${\hat{y}}_{k}$ represent the soft label and the model predicted probability for the *k*-th sub-label, respectively. This loss function optimizes all sub-labels equally. This mechanism drives the model to simultaneously seek common and specific features in the feature space that characterize anomalies in different flow rate segments of the small airways.

### 2.4. Model Evaluation

To enhance the robustness of the performance evaluation, this study adopted a five-fold cross-validation strategy. The dataset was randomly partitioned into five mutually exclusive subsets. We alternately selected one subset as the test set, while the remaining four served as the training set. The final reported model performance is the average of the results from these five independent tests.

We constructed a hierarchical evaluation framework to test the model from two dimensions. The first dimension is SAD identification, which constitutes the core work of this study. We compared the SAD identification results output by the voting module with the ground-truth labels to evaluate the final performance. The second dimension is sub-label identification. To enhance the interpretability of the model and provide more information for clinical practice, we independently evaluated the identification ability of the model for three sub-labels, FEF_25–75_, FEF_50_, and FEF_75_.

During the inference evaluation phase, we binarized the predicted probabilities output by the Sigmoid function using a threshold of τ=0.5 to calculate standard classification metrics. This evaluation framework not only verifies the overall identification capability of the model but also facilitates our understanding of how the model makes final decisions by learning the detailed features of SAD. Regarding the selection of quantitative metrics, we selected four metrics that are most commonly used in medical identification tasks, including Accuracy, Sensitivity, Specificity, and F1-score.

## 3. Experiments and Results

### 3.1. Dataset Construction

#### 3.1.1. Data Collection

The data utilized in this study were collected from the China–Japan Friendship Hospital. The record for each subject comprises three distinct data components. The first component encompasses demographic characteristics, specifically age, sex, height, and weight. The second component consists of raw time-series signals acquired via the IOS device. These signals are the continuous oral pressure and airflow waveforms recorded during the testing period. The third component includes results from spirometry, specifically indices reflecting small airway function such as FEF_25–75_, FEF_50_, and FEF_75_. These indices were utilized to construct data labels in accordance with clinical standards. To ensure the internal consistency and reliability of the dataset, all data acquisition was performed by professional technicians. This process strictly adhered to the standardization guidelines established by the American Thoracic Society and the European Respiratory Society (ATS/ERS) [32,33]. The experimental protocol received approval from the Ethics Review Committee of the China–Japan Friendship Hospital.

We included subjects who completed IOS and spirometry during the same visit. Both tests had to meet ATS/ERS guideline-based quality control requirements, ensuring acceptable coherence levels and absence of significant artifacts. Essential variables and raw IOS signals required for model construction had to be available. We excluded records with unacceptable test quality, missing essential signals or variables, or other predefined data integrity issues. When repeated examinations were available for the same subject, we applied a consistent rule to avoid duplication and retained only one eligible record per subject. The dataset was derived from deidentified pulmonary function testing records. Given the retrospective nature of the dataset, detailed clinical metadata such as specific phenotypes were not systematically recorded for all subjects.

Ultimately, a total of 2510 valid samples were included in this study. The dataset consists of 1061 SAD samples and 1449 non-SAD samples according to clinical criteria. The demographic distribution and baseline pulmonary function characteristics of the dataset are detailed in Table 1.

Regarding statistical presentation, continuous variables are expressed as mean ± standard deviation, while categorical variables are presented as frequencies and percentages. Given that certain physiological parameters exhibited non-normal distribution characteristics, the Mann–Whitney U test was used for between-group comparisons of continuous variables to ensure analytical robustness [34,35]. For categorical variables, the Chi-square test was utilized. A two-sided *p*-value of less than 0.05 was considered statistically significant.

#### 3.1.2. Data Preprocessing

To ensure data consistency for the deep learning model and reduce dimensional mismatch between different branches, we implemented a standardized data preprocessing workflow.

Regarding the TFRIM data, we first removed invalid frequency bins with coherence $\gamma^{2}<0.9$. Among the remaining bins, we retained the 24 frequency channels with the highest coherence for each sample, so that each sample was processed into a tensor $x_{\mathrm{TFRIM}}\in R^{C_{TF}\times H_{TF}\times W_{TF}}$ with fixed dimensions. In this configuration, $C_{TF}=2$ corresponds to the resistance and reactance channels, $H_{TF}=24$ denotes the highest-coherence frequency channels retained after filtering, and $W_{TF}=48$ represents the number of time windows. This standardized tensor serves directly as the input for the TFRIM encoding branch. Notably, this coherence-based filtering and top-24 frequency channel selection are performed independently for each sample using only its own pressure–flow signals, without relying on any dataset-level statistics or label information.

Regarding the time-series data, for each subject we formatted the raw one-dimensional pressure and flow sequences into tensors $x_{\mathrm{TIME}}$ with a fixed length of $L_{\mathrm{time}}=9120$ sampling points to ensure temporal alignment with the TFRIM. This length precisely covers the time span corresponding to the TFRIM generation, thereby ensuring that the dual-branch network processes physiological information originating from the same respiratory period.

Furthermore, Z-score normalization was applied to all input data to standardize feature scales. To avoid data leakage, a strict data leakage prevention strategy was implemented. The normalization calculation is defined as:

(22) $$x_{\mathrm{norm}}=\frac{x-\mu_{\mathrm{train}}}{\sigma_{\mathrm{train}}}$$

Specifically, for each feature channel, the mean $\mu_{train}$ and standard deviation $\sigma_{train}$ were calculated exclusively using the training set. These statistical parameters were subsequently frozen and applied to the normalization of the test set. This approach effectively prevents the statistical distribution information of the validation set from leaking into the model during the preprocessing stage.

#### 3.1.3. Label Definition

This study implements a two-level label construction scheme for model training and inference. Specifically, soft labels are introduced during training to provide richer supervisory information, whereas hard binary labels are used to produce the final SAD or non-SAD decision during inference.

For the construction of binary hard labels, denoted as $y_{\mathrm{SAD}}$, we adhered to clinical expert consensus and relevant guidelines to establish the ground truth for this study [4,36,37,38]. Here, $y_{\mathrm{SAD}}\in \left\{0,1\right\}$ denotes the final binary SAD label at the subject level. The determination of these labels relies primarily on three key indicators reflecting peripheral airway function: FEF_25–75_, FEF_50_, and FEF_75_. While other intermediate flow points could theoretically be extracted, we restricted our analysis to these widely adopted indices to ensure strict alignment with established reference values and routine diagnostic workflows. We defined the ratio of the measured value to the predicted value for these three indicators as $v_{k}$, where k∈{1,2,3} corresponds to each respective indicator. According to clinical standards, an indicator is considered abnormal if $v_{k}<0.65$. To mitigate potential assessment instability caused by fluctuations in individual indicators, we followed the clinical rule stipulating that a sample is labeled as SAD ($y_{\mathrm{SAD}}=1$) if at least two of the three indicators are deemed abnormal; otherwise, it is labeled as non-SAD ($y_{\mathrm{SAD}}=0$).

Considering that traditional binary hard labels may not fully capture the fine-grained distributional information of the samples, we designed a soft label generation strategy, denoted as $y_{\mathrm{soft}}^{\left(k\right)}$ for each subtask during training. These soft labels provide richer supervision signals for the three subtasks during training than binary hard labels. The calculation is defined as follows:

(23) $$y_{\mathrm{soft}}^{\left(k\right)}=\sigma \left(\lambda \cdot \left(v_{\mathrm{th}}-v_{k}\right)\right)$$

In this formula, $y_{\mathrm{soft}}^{\left(k\right)}\in \left(0,1\right)$ represents the soft label for the *k*-th subtask, and σ(·) denotes the standard sigmoid function. The variable $v_{k}$ is the ratio of the measured to the predicted value for the corresponding indicator, and $v_{\mathrm{th}}$ is set to the clinical evaluation threshold of 0.65. To control the smoothness of the transition near the threshold, we introduced a scaling factor λ, which was empirically set to 20. This mapping mechanism $y_{\mathrm{soft}}^{\left(k\right)}\to 1$ when $v_{k}$ is well below $v_{\mathrm{th}}$, $y_{\mathrm{soft}}^{\left(k\right)}\to 0$ when $v_{k}$ is well above $v_{\mathrm{th}}$, and $y_{\mathrm{soft}}^{\left(k\right)}$ approach 0.5 when $v_{k}\approx v_{\mathrm{th}}$, thereby retaining uncertainty near the clinical cutoff. This design aims to encourage the model to learn the gradual changes in features associated with the deterioration of physiological indicators. It is important to emphasize that the labels in this study were constructed using spirometry indicators to train the model to identify SAD as defined by spirometry.

### 3.2. Implementation Details

All the experiments were performed on a workstation equipped with a 12-core Intel(R) Xeon(R) Silver 4214R CPU @ 2.40 GHz, 90 GB of RAM, and an NVIDIA GeForce RTX 3080 Ti GPU, running the Ubuntu 20.04 operating system.

The model parameters were optimized using the Adam optimizer with an initial learning rate of $1\times 10^{-4}$. A cosine annealing learning-rate schedule was adopted to improve optimization and convergence. The batch size was set to 256. Training was terminated when the validation loss no longer decreased. All the models were implemented based on PyTorch, version 2.1.1.

### 3.3. Experimental Settings

#### 3.3.1. Comparative Experiments

To assess the effectiveness of the proposed model and the contribution of its architectural design in the SAD identification task, we selected representative algorithms covering both traditional machine learning and mainstream deep learning architectures as comparative baselines. It should be noted that to ensure a fair comparison, all baseline methods were re-implemented and trained under identical dataset partitioning and preprocessing conditions.

To verify the advantages of the proposed end-to-end deep learning model over traditional methods, we employed the XGBoost algorithm [39] as a representative machine learning baseline. In this setup, the input features comprised demographic characteristics and six clinically common IOS parameters. Specifically, these parameters included airway resistance at 5 Hz, 20 Hz, and 35 Hz, airway reactance at 5 Hz, resonant frequency, and airway impedance at 5 Hz. To adapt to the multi-label task defined in this study, we constructed three independent XGBoost classifiers. These classifiers were tasked with identifying FEF_25–75_, FEF_50_, and FEF_75_, respectively. The final SAD determination was generated through a voting mechanism. Additionally, hyperparameters were optimized via grid search.

Subsequently, to investigate the influence of convolutional network depth on IOS feature extraction, we constructed a ResNet group. This group included three variants, ResNet-10, ResNet-18, and ResNet-34. Both ResNet-18 and ResNet-34 adopted the standard architecture proposed by He et al. [40]. Furthermore, given that small-sample medical datasets can lead to overfitting in deep networks, we designed a lightweight ResNet-10 model. This model was used to evaluate performance across different parameter scales. The ResNet-10 variant retained the residual connection mechanism but reduced the number of convolutional blocks in each stage to a configuration of [1, 1, 1, 1]. This design aimed to achieve an optimal balance between model capacity and generalization ability. We replaced all convolutional layers with 1D convolutions to accommodate the time-series input data. Additionally, we also introduced the classic GoogLeNet as a baseline [41]. The purpose of using this model was to assess the effectiveness of multi-scale feature extraction strategies in capturing respiratory impedance characteristics. Similar to the previous models, its internal structure was adapted to a 1D convolutional format for our implementation.

Finally, to evaluate the capability of self-attention mechanisms in capturing long-range dependencies within IOS signals, we implemented a standard Transformer encoder [42]. Due to constraints on data size, we compressed the model parameters to prevent severe overfitting. Specifically, the number of encoder layers was set to $L_{t}=3$, the hidden layer dimension to $d_{\mathrm{trans}}=256$, and the number of multi-head attention heads to $N_{\mathrm{head}}=4$. In this baseline, the IOS sequence was first sliced into multiple patches. These patches were then augmented with positional encodings before serving as input to the network.

#### 3.3.2. Ablation Studies

To validate the effectiveness of the proposed dual-domain complementary deep learning framework, we designed a set of ablation experiments. All experiments were conducted under identical hyperparameter settings and computational environments, with controlled adjustments applied exclusively to the input features. The proposed model integrates TFRIM, time-series signals, and demographic characteristics. To quantify the specific contributions of these three data and their combinations to SAD identification performance, we conducted seven sets of experiments for systematic evaluation.

First, we evaluated single-branch inputs by independently training the TFRIM encoding branch, the time-series encoding branch, and the demographic encoding branch. The extracted features were fed into classifiers with identical structures. Specifically, for the experiment utilizing only demographic features, we employed an MLP to directly process the feature vectors for the identification task.

Next, we evaluated the performance of three dual-branch combinations. These included TFRIM combined with the time-series branch, TFRIM combined with demographic characteristics, and the time-series branch combined with demographic characteristics. In these experiments, we removed the network modules corresponding to the missing input. We also adjusted the input dimensions of the feature fusion layers accordingly.

Finally, we evaluated the performance of the complete model incorporating all input data.

Furthermore, to examine whether incorporating demographic features via the proposed DAFM improves fusion performance, we compared four fusion mechanisms under the full-input setting. These mechanisms were Concatenation, Gating, Additive Bias, and DAFM.

The first baseline is Concatenation Fusion. This is the most straightforward approach. First, we extracted high-dimensional features, denoted as $h_{d}$, from the demographic features d via an MLP. Subsequently, we directly concatenated $h_{d}$ with the backbone fused features $x_{\mathrm{fused}}$ along the channel dimension. This concatenation-based fusion serves as a commonly used early-fusion baseline in multimodal learning [43]. The combined features were then fed into the classifier. The formula is defined as:

(24) $$x_{\mathrm{out}}=\mathrm{Concat}\left(x_{\mathrm{fused}},h_{d}\right),\;\mathrm{where}\;h_{d}={\mathrm{MLP}}_{h}\left(d\right)$$

The second method is Gating Fusion. This strategy maps the demographic features d to a gating coefficient g via an MLP. This coefficient lies within the interval (0,1). We then used g to weight the backbone fused features for integration. This formulation is inspired by gating-based fusion mechanisms widely adopted in multimodal representation learning [44]. The calculation is as follows:

(25) $$x_{\mathrm{out}}=x_{\mathrm{fused}}⊙g,\;\mathrm{where}\;g=\sigma \left({\mathrm{MLP}}_{\mathrm{gate}}\left(d\right)\right)$$

The third method is Additive Bias Calibration Fusion. This approach assumes that demographic features primarily serve as an additive bias. Consequently, we generated a bias feature vector b from the demographic features d using an MLP. This additive bias modulation can be viewed as a simplified form of feature-wise conditioning used in prior work [45]. This vector was added directly to the backbone features. The equation is:

(26) $$x_{\mathrm{out}}=x_{\mathrm{fused}}+b,\;\mathrm{where}\;b={\mathrm{MLP}}_{\mathrm{bias}}\left(d\right)$$

Finally, we evaluated the DAFM Fusion strategy proposed in this study. This corresponds to the scheme defined in Equation (19). Unlike the previous methods, DAFM introduces both a scaling factor α and a shifting factor β. This allows for a more flexible calibration through affine transformation. Mathematically, this method encompasses the properties of both multiplicative gating and additive bias mechanisms. Therefore, it theoretically possesses more flexible feature calibration capabilities. Furthermore, the value range of the scaling factor α is not limited to (0,1). Instead, it extends to the entire real number domain. This enables more flexible feature scaling operations. The formula is:

(27) $$x_{\mathrm{out}}=\alpha ⊙X_{fused}+\beta ,\;\mathrm{where}\;\left[\alpha ,\beta \right]=\mathrm{MLP}\left(d\right)$$

### 3.4. Results

#### 3.4.1. Results of Main Task: SAD Identification

The average performance metrics derived from five-fold cross-validation were adopted as the final evaluation results, as detailed in Table 2. The proposed model achieved an overall accuracy of 0.8139 on the current dataset. Notably, the model demonstrated a specificity of 0.8724, indicating a strong capability to exclude non-SAD cases and suggesting potential utility in clinical screening scenarios. Furthermore, the precision score of 0.8186 reflects the reliability of positive predictions, while the sensitivity of 0.7340 and F1 score of 0.7687 indicate a favorable balance between sensitivity and precision.

To validate the effectiveness of the proposed dual-domain complementary deep learning framework, comparative experiments were conducted against classic machine learning algorithms and various mainstream deep learning baselines. These comparative experiments were conducted under identical settings, and the quantitative results are detailed in Table 3.

Firstly, the XGBoost model, representing traditional machine learning, exhibited limited performance, recording the lowest accuracy of 0.6926 and sensitivity of 0.4834. This limitation may be attributable to the reliance on static and discrete IOS-derived parameters, which may be insufficient to fully capture the dynamic temporal characteristics of airway impedance during respiration.

Secondly, we analyzed the impact of network depth on performance using the ResNet series. The results revealed a non-monotonic trend. The shallow ResNet10 network showed signs of underfitting, which was likely due to constrained feature extraction capabilities. Conversely, although ResNet34 possesses a larger theoretical receptive field, it exhibited performance degradation. This decline appeared to be caused by overfitting given the current dataset size, suggesting that excessively deep architectures may not be optimal for this specific task. Among the series, ResNet18 achieved the best balance between parameter quantity and feature extraction, reaching an accuracy of 0.7291.

Overall, the proposed model outperformed the aforementioned baselines across all evaluated metrics. Notably, our method achieved performance gains even when compared to the Transformer, which was the strongest baseline. This advantage may be attributable to the integration of TFRIM spectrograms with the dual-stream architecture. By preserving the time-domain details of the original signal while effectively leveraging dynamic frequency-domain texture features, our approach realized superior SAD identification capabilities.

We conducted a series of ablation studies to evaluate the specific contributions of different input branches to the SAD identification task and to validate the effectiveness of the multi-branch fusion strategy. These experiments covered various combinations, ranging from single-branch inputs to joint multi-branch inputs. Detailed performance metrics are presented in Table 4.

First, in the single-branch setting, utilizing only the raw time-series signal or the TFRIM yielded accuracies of 0.7430 and 0.7490, respectively. These results suggest that each individual branch contains certain discriminative patterns. However, single-branch feature extraction appears insufficient to fully capture the complex pathophysiological changes associated with SAD. Furthermore, relying solely on demographic features resulted in performance only slightly above chance level. This implies that demographic features do not directly reflect airway pathological features. Instead, they likely serve as auxiliary calibration information rather than a primary identification basis.

Upon introducing a second input branch, performance improvements were consistently observed. Specifically, combining the raw time-series signal with the TFRIM increased accuracy. This improvement indicates potential complementarity between time-domain respiratory dynamics and time–frequency impedance spectral texture features. Consequently, their combination provides a more comprehensive characterization of airway function. Additionally, incorporating demographic features into either the time-series or TFRIM inputs yielded a performance gain of approximately 2%. This finding supports the efficacy of the proposed DAFM module. It suggests that integrating demographic information reduces the confounding effects of inter-individual variability on SAD identification.

This indicates that the three input branches likely form a synergistic interaction within the feature space. In this synergy, the raw signal provides respiratory dynamics, the TFRIM contributes time–frequency texture information, and demographic features offer global calibration. Such a fusion strategy appears instrumental in enhancing the accuracy of SAD identification.

To evaluate the effectiveness of the proposed DAFM fusion scheme, we compared it with three classical feature fusion strategies. The experimental results are detailed in Table 5. As a baseline, the concatenation fusion strategy directly joins demographic features with the feature representation $X_{fused}$. Although this approach introduces auxiliary information, it relies on simple dimensional stacking. Consequently, it may fail to explicitly capture the specific influence of demographic factors on airway impedance.

The gating fusion strategy performed slightly worse than the concatenation approach. This phenomenon suggests that using only a multiplicative gating mechanism to adjust feature amplitude may be insufficient. Mathematically, it might not effectively characterize the additive shift effect that demographic features exert on the respiratory impedance baseline.

In contrast, the additive bias calibration fusion strategy achieved higher accuracy than both the concatenation and gating methods. This result supports the hypothesis that demographic features primarily serve a baseline correction role in physiological signal processing. It appears that explicit bias calibration effectively guides the model to adapt to distributional differences among individuals.

Finally, the proposed DAFM module demonstrated the most favorable performance among the evaluated methods. This module treats demographic features not as isolated information fragments but as active calibration signals. By mapping these features to adaptive modulation parameters, the DAFM module achieves dynamic, sample-level modulation of airway-impedance features. Functionally, this mechanism can be viewed as analogous to the clinical process where physicians adjust decision thresholds based on patient physique. Therefore, this approach can reduce the confounding effects of inter-individual physiological variability on SAD identification.

#### 3.4.2. Results of Subtasks

To further analyze the performance characteristics of the model, we evaluated its ability to identify abnormalities in three key small airway function indices. These indices include FEF_25–75_, FEF_50_, and FEF_75_.

Table 6 quantifies the model’s performance across individual subtasks. The final decision is obtained by combining subtask predictions via the voting mechanism. In this setting, the subtasks exhibit complementary characteristics across metrics, potentially benefiting overall identification performance. Specifically, the model exhibited high sensitivity (0.8446) in the task of identifying FEF_75_ abnormalities. This suggests that the network is capable of capturing feature patterns associated with distal airway flow limitations. Consequently, this capability helps reduce the rate of missed detections for potentially abnormal samples. Conversely, the model demonstrated high specificity (0.9079) when identifying FEF_50_ abnormalities. This implies that the model adopts a more conservative approach in assessing mid-airway function. Therefore, it exhibits high reliability in excluding false positive cases.

These distinct, complementary performance characteristics support the rationale of the multi-label voting mechanism introduced in this study. This mechanism effectively integrates the capability of the FEF_75_ classification head to capture abnormal samples with the ability of the FEF_50_ classification head to suppress false alarms.

Through this decision-level fusion, the final SAD identification results achieved a favorable balance between sensitivity (0.7340) and specificity (0.8724). This strategy not only surpasses the average performance of individual metrics but also contributes to enhancing the robustness of the identification system in clinical application scenarios.

**Figure 4:**
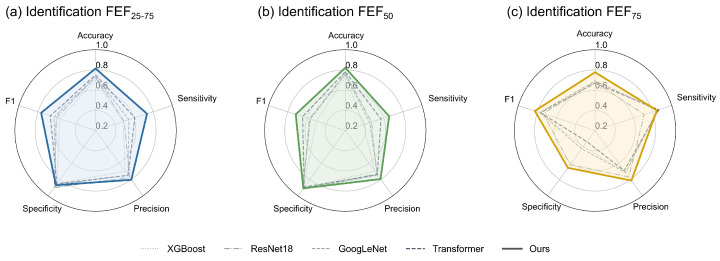
Radar–chart comparison of subtask identification performance between the proposed model and mainstream baselines.

Figure 4 further presents the performance comparison between the proposed model and existing mainstream baselines on these proxy subtasks using a radar chart. As illustrated, the proposed model encloses a larger area across the evaluation dimensions compared to the baseline models. This observation suggests that our approach yields a more balanced overall performance profile.

Notably, the FEF_75_ index, which reflects distal airway function, typically presents a greater detection challenge. On this specific metric, both XGBoost and Transformer exhibited certain performance limitations. In contrast, the proposed architecture maintained relatively high recognition accuracy. This robustness is likely attributed to the rich time–frequency texture information provided by the TFRIM, as well as the adaptive calibration capability of the DAFM module. These results imply that the model possesses potential efficacy in capturing subtle airway impedance abnormalities.

### 3.5. Model Interpretability and Feature Visualization

**Figure 5:**
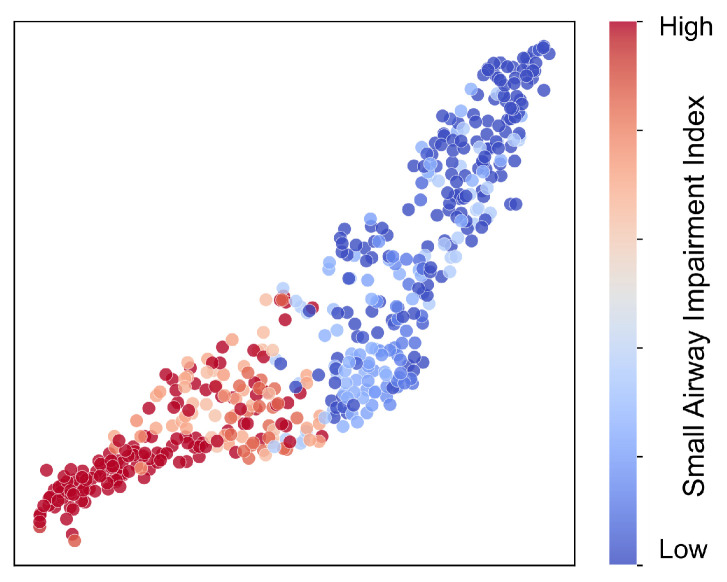
t-SNE projection of learned feature embeddings.

To provide an intuitive analysis of the high-dimensional feature distribution structure learned by the model, we extracted feature vectors from the penultimate layer. Subsequently, we employed the t-SNE algorithm to project these vectors into a two-dimensional space, as illustrated in Figure 5. In this visualization, each data point represents an individual subject.

To clearly depict the distributional trend ranging from normal to impaired small airway function, we defined a Small Airway Impairment Index, denoted as *S*. This index integrates three soft labels into a single continuous variable. Specifically, drawing upon voting principles commonly used in clinical decision-making, we designed a differentiable fusion function to calculate *S*:

(28) $$S=y_{\mathrm{soft}}^{\left(1\right)}y_{\mathrm{soft}}^{\left(2\right)}+y_{\mathrm{soft}}^{\left(2\right)}y_{\mathrm{soft}}^{\left(3\right)}+y_{\mathrm{soft}}^{\left(1\right)}y_{\mathrm{soft}}^{\left(3\right)}-2y_{\mathrm{soft}}^{\left(1\right)}y_{\mathrm{soft}}^{\left(2\right)}y_{\mathrm{soft}}^{\left(3\right)}$$

This formulation is designed such that the *S* value yields a higher response when two or more indicators fall below their respective thresholds. This characteristic maintains consistency with clinical decision-making rules. For visualization purposes, we mapped the color coding to the *S* value, achieving a smooth visual transition from normal small airway function (represented by deep blue) to higher degrees of impairment (represented by deep red). An *S* value approaching 1 indicates that the three indicators are simultaneously or largely below the threshold, suggesting a higher risk of small airway impairment. Conversely, an *S* value approaching 0 implies that the indicators are generally above the threshold, suggesting normal airway function. It is crucial to emphasize that *S* is an engineering-based continuous proxy constructed from spirometry thresholds. Its primary purpose is to facilitate visualization and feature structure analysis; therefore, it is not equivalent to a strict clinical severity grading system.

Observing the visualization of the feature space, the samples collectively exhibit a continuous band-like distribution pattern accompanied by a distinct color gradient. The deep blue cluster representing the healthy state and the deep red cluster representing the impaired state occupy opposite ends of the trajectory. Samples in the intermediate region demonstrate a gradual transition from healthy to transitional and then to impaired states, with slight overlapping. This distributional morphology presents a trend consistent with the definition of *S*. This phenomenon suggests that the feature representation likely encodes information regarding the deviation of small airway-related indicators from their thresholds.

**Figure 6:**
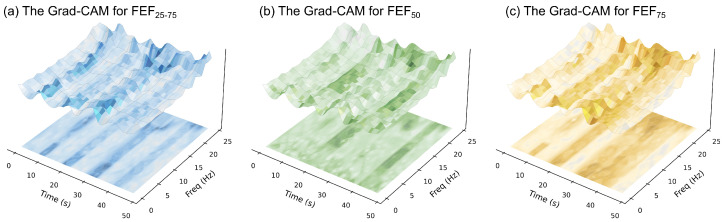
Grad–CAM–based 3D fusion visualization of model attention on TFRIM.

We employed Grad-CAM to analyze the time–frequency texture features from the TFRIM branch. This analysis aimed to interpret the features prioritized by the model. Figure 6 presents a 3D fusion visualization. It intuitively maps the model’s focus onto the TFRIM. The *z*-axis height represents the TFRIM amplitude. Meanwhile, the surface color indicates the distribution of visual attention. Darker colors correlate with a higher degree of model attention.

Although the subtasks target different indices, the model consistently exhibits respiratory phase selectivity. Specifically, the model concentrates on the expiratory phase window. This aligns with the pathophysiological mechanisms of SAD. Abnormal impedance fluctuations occur primarily during exhalation. These manifest as flow limitations [46,47]. The model appears to suppress inspiratory background signals and focus on end-expiratory dynamics. This suggests the network may capture transient pathological textures associated with airway collapse.

Furthermore, in the frequency dimension, highlighted regions are concentrated within specific bands. This indicates the network identifies respiratory phases and captures non-linear impedance characteristics. We further observed distinct attention patterns across different subtasks. This pattern suggests that the model may encode subtask-specific information related to airway function. For the FEF_75_ and FEF_25–75_ tasks (reflecting small airway function), attention focuses on the low-frequency band. This distribution is consistent with the physics of IOS. According to oscillation mechanics, only low-frequency waves penetrate deep into the lungs. Therefore, variations in this band may reflect the peripheral small airway state. In contrast, for the FEF_50_ task (reflecting mid-airway function), attention extends to a mixed low-to-mid frequency range.

This spectral differentiation is consistent with the frequency-dependent characteristics of IOS signals. The Grad-CAM results suggest that the discriminatory basis is not uniformly distributed. Instead, it exhibits differentiated attention to low-to-mid frequency information. This alignment with expected signal-sensitive bands supports the interpretability of the model’s performance.

**Figure 7:**
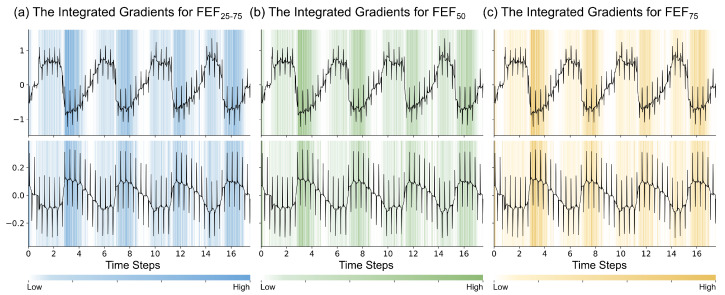
Integrated Gradient attribution visualization for raw IOS time-series signals.

To further investigate the attribution focus of the time-series branch during feature extraction, we employed the Integrated Gradients algorithm to calculate the time-step attribution values of the time-series signals. Figure 7 visualizes the attribution for raw IOS signals across three subtasks. The background color intensity represents the attribution of each signal point.

Observing the attribution distribution reveals patterns at both macro and micro levels. On a macro scale, high-attribution regions are synchronized with the respiratory cycle. Specifically, attribution values concentrate in the expiratory phase. In contrast, attribution values during the inspiratory phase are lower. This suggests that the model is sensitive to dynamic airway compression. It aligns with the expiratory flow limitation observed in SAD patients.

On a micro scale, the model exhibits selectivity for specific signal components. Input signals contain large-amplitude, low-frequency breathing waves. However, the model assigns low attribution values to these broad contours. Instead, high-frequency oscillatory impulses receive higher attribution values. As shown in Figure 7, high attribution values cluster at the points where IOS pulses are applied. This implies the network utilizes external oscillatory signals for feature extraction. It does not merely memorize the breathing morphology.

**Figure 8:**
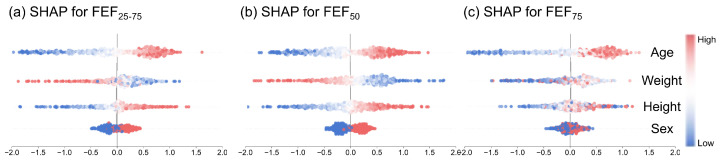
SHAP summary plots of demographic feature contributions to subtask predictions.

To assess how the DAFM module utilizes demographic information for result calibration, we employed the SHapley Additive exPlanations (SHAP) method to calculate the marginal contribution of each demographic feature to the model’s output. Figure 8 presents the SHAP summary plots for the three subtasks (identifying abnormalities in FEF_25–75_, FEF_75_, and FEF_50_). In these plots, each row represents a feature, and each dot represents an individual data sample. The color of the dot indicates the magnitude of the feature value, while the horizontal position denotes the direction and intensity of the contribution to the prediction. A positive value indicates an increased probability of SAD identification, whereas a negative value implies the opposite.

The distribution of SHAP values reveals a consistent pattern of feature importance across different subtasks. Among all features, age consistently exhibits the highest importance, with its SHAP values spanning the widest range. Specifically, samples representing older age (indicated in red) are predominantly concentrated in the positive SHAP value region. This suggests that advancing age positively drives the model’s determination of airway dysfunction. This finding aligns with established knowledge in respiratory physiology, which notes that pulmonary function undergoes a physiological decline with age, thereby increasing the baseline probability of pathology.

Weight and height follow in importance, exhibiting substantial contributions to the model output across the subtasks. This reflects the corrective effect of body physique parameters on the feature distribution. In contrast, the marginal contribution of gender is relatively small, with its SHAP values converging near zero. Nevertheless, with males encoded as 1, a slight tendency toward SAD identification for male subjects is observable. This data preference offers interpretability from an epidemiological perspective. It may implicitly reflect the statistically higher risk of small airway dysfunction in the male population, potentially due to factors such as higher smoking rates or occupational exposure.

In summary, the SHAP analysis suggests that the DAFM module has acquired a calibration logic consistent with clinical priors. Specifically, the model appears to dynamically adjust decision boundaries based on the subject’s age and physique characteristics. This feature attribution pattern, which aligns with existing clinical theories, further supports the validity and interpretability of this branch as a personalized calibration unit within the multi-modal network.

The visualization analyses suggest internal consistency in the model’s decision rationale. Although the TFRIM and raw time-series branches operate on distinct representations, their attribution patterns are largely concordant. Specifically, both Grad-CAM and Integrated Gradients consistently assign higher relevance to the expiratory phase and low-frequency oscillatory components. This cross-method agreement, together with the continuous embedding gradient observed in the t-SNE projection and the demographic weighting patterns revealed by SHAP, provides convergent evidence that the model leverages physiologically plausible airway dynamics, thereby reducing the likelihood that the performance is dominated by background noise or spurious correlations.

## 4. Discussion

The primary challenge in IOS-based SAD identification lies in the subtle and time-varying signatures of small airway dysfunction. These characteristics are often obscured during the global averaging process inherent in traditional static parameter calculations. Consequently, conventional methods relying on discrete scalar parameters such as R5 and X5 are limited by temporal information compression. These methods struggle to capture critical dynamic changes within the respiratory cycle. To overcome this limitation, we developed a dual-domain complementary deep learning architecture that integrates joint time–frequency information, rather than relying solely on static scalar parameters. Under this framework, the resulting model achieves a clear improvement over the XGBoost baseline, with an accuracy of 0.8139 compared to 0.6926, supporting the motivation for moving beyond static scalar parameters. This outcome suggests that high-dimensional raw time-series signals contain discriminative information that low-dimensional scalars cannot fully represent. Furthermore, ablation studies provide insight into the mechanism underlying this performance improvement. The ablation results suggest that the joint modeling of TFRIM and raw time-series signals provides a complementary effect. This combined approach appears superior to using single-branch inputs alone. Specifically, the CNN branch focuses on extracting time–frequency impedance texture features of the impedance distribution. In parallel, the Mamba branch captures long-range temporal dependencies and transient waveform features within the respiratory signals. This dual-branch design effectively integrates time–frequency impedance texture features with temporal waveform features. Such integration achieves a multi-dimensional characterization of lung function. Consequently, the proposed architecture facilitates the identification of complex features associated with SAD.

It is important to clarify the methodological scope of this study. The reference labels were derived from spirometric indices. Therefore, our goal is not to redefine SAD or to replace spirometry as a diagnostic reference standard. Instead, we aim to establish a cross-modality mapping between tidal-breathing IOS measurements and spirometry-defined SAD criteria. This study tests whether IOS-based physiological features can approximate spirometry-defined decision criteria without requiring forced expiratory maneuvers. This property is clinically relevant because forced maneuvers are challenging for certain populations and may be difficult to implement in some practice settings.

The results support the feasibility of an IOS-based assessment that approximates spirometry-defined SAD classification. This provides a complementary option in situations where spirometry cannot be performed with adequate quality. Nevertheless, the current framework is anchored to spirometry-derived labels and therefore demonstrates spirometry-consistent prediction rather than superiority over spirometry or independent clinical reference standards.

The dual-stream network extracts features reflecting airway status from IOS signals. However, the absolute values of respiratory impedance are influenced by the physiological background of the subject. Natural variations in lung function baselines exist due to age, height, weight, and gender. Consequently, using uncalibrated features for identification can introduce significant inter-individual representation differences. This variability makes it difficult to apply a unified standard for SAD assessment. To mitigate this issue, the DAFM module integrates demographic features as modulation parameters rather than treating them merely as ordinary input features. Specifically, this module learns physiological patterns across populations to generate adaptive scaling factors and shifting factors. These factors are then used to dynamically calibrate the extracted physiological features. This mechanism is conceptually consistent with the calibration logic used in clinical lung function assessment. This design can help suppress interference arising from inter-individual physiological differences. As a result, the model may focus more on identifying impedance anomalies caused by pathological changes. Comparative experiments indicate that DAFM outperforms simple concatenation and gating fusion, and is also superior to an additive-bias calibration fusion baseline. This finding suggests that the role of demographic features lies primarily in providing global feature distribution calibration rather than local feature selection. SHAP analysis results support this interpretation. SHAP analysis assigns the largest contribution to age, followed by weight and height. This pattern is consistent with general physiological knowledge, suggesting that the model may adjust decision thresholds with respect to aging and body habitus [48]. Ultimately, this adaptive calibration may improve identification accuracy across populations with diverse physiological characteristics.

Beyond improvements in quantitative metrics, evaluating the clinical utility of a model also requires assessing whether its decision rationale is physiologically plausible. In this study, visualization analyses suggest that the feature extraction patterns learned by the model are broadly consistent with principles of respiratory mechanics.

Integrated Gradient attributions on the time-series input suggest that the model places greater emphasis on the expiratory phase. This occurs even without explicit respiratory phase annotations. The resulting attribution pattern is consistent with expiratory flow limitation. Such limitation may be related to reduced elastic recoil in small airways in SAD [46,47]. Overall, these findings suggest that the classifier may rely more on clinically relevant patterns. They also suggest a reduced reliance on spurious correlations driven by noise or potential confounders.

In the frequency domain, Grad-CAM maps on the TFRIM show that activation hotspots tend to concentrate in the low-frequency region. This pattern is observed when the model predicts FEF_75_ and FEF_25–75_. These are spirometric indices reflecting peripheral airway function. The observation is consistent with a general principle in forced oscillation. Lower-frequency oscillations tend to probe more distal airways. They can also be more informative for distal airway impedance [26]. In contrast, a different distribution is observed for FEF_50_. FEF_50_ is often considered to reflect more central-to-mid airway function. For this index, the activation hotspots tend to extend toward the mid-frequency band. Taken together, this frequency-dependent pattern suggests that the model may leverage distinct frequency components. These components may help differentiate information across airway levels.

Furthermore, the t-SNE projection suggests a more continuous structure in the latent space. The structure spans from healthy to impaired states. It does not form two clearly separated clusters. This arrangement qualitatively mirrors the trend of the small airway impairment index used in this study. Collectively, these results support the interpretation that the learned representations may capture continuous pathological variation to some extent. This provides a basis for exploring continuous stratification. It also motivates future work on disease severity modeling.

The proposed framework can be integrated into routine pulmonary function testing as an adjunct decision-support module. After IOS acquisition during tidal breathing, the model can be executed automatically to output a binary prediction of SAD that is aligned with spirometry-derived reference criteria. Such a binary output may support triage and follow-up decisions, particularly in scenarios where forced expiratory maneuvers are difficult to perform with adequate quality. In practice, the predicted label can be summarized in the test report together with the accompanying interpretability results, which may facilitate clinical review and improve transparency. From a translational perspective, the present study establishes the feasibility of IOS-based SAD identification within a standardized testing workflow. Future work will focus on prospective validation across centers/devices and on defining operating procedures for deployment.

While this study demonstrates the potential of IOS-based SAD identification, several limitations warrant consideration. First, the current evidence is based on a cohort with limited diversity and primarily internal validation; although five-fold cross-validation provides a robust internal estimate, it cannot replace evaluation on geographically or clinically distinct external cohorts. Therefore, the generalizability of the model across centers, devices, and populations remains to be confirmed through independent external datasets. Second, due to the retrospective nature of the dataset, detailed clinical metadata were not universally available, precluding etiology-specific subgroup analyses. Consequently, the model is formulated as a binary classifier focused on functional identification, which does not yet provide severity grading or distinguish specific disease phenotypes. Third, from a deployment perspective, the current architecture still entails a nontrivial computational footprint, and further optimization is needed for efficient operation on resource-constrained portable or embedded platforms.

To address these limitations, future work will prioritize expanding data collection and validation, including multi-center and multi-device cohorts and external testing to better assess robustness and transportability. We also plan to extend the task beyond binary identification by developing more fine-grained modeling for severity grading and phenotype-related stratification, leveraging the continuous structure suggested by representation analyses. In parallel, we will explore lightweight architectures and compression strategies to evaluate the feasibility of near-real-time edge inference on embedded systems, supporting practical integration into point-of-care screening workflows.

## 5. Conclusions

This study presents a dual-domain complementary deep learning framework based on IOS. By integrating time–frequency impedance patterns with temporal waveform features, the proposed method moves beyond simple static parameter analysis to enable a richer characterization of pulmonary function dynamics. Additionally, the incorporation of the DAFM module aims to reduce discrepancies in learned representations arising from physiological baseline variations, which may improve robustness across subjects with varying baseline characteristics.

Experimental evaluations indicate that the model achieves competitive performance relative to traditional machine learning approaches and selected representative deep learning baselines. Furthermore, multi-dimensional interpretability analyses suggest that the model’s decision rationale is broadly consistent with principles of respiratory mechanics. This observation supports the plausibility of the learned features from a respiratory-mechanics perspective. In conclusion, this study offers a promising approach for SAD identification that requires reduced patient effort, potentially facilitating noninvasive screening in primary care settings and among populations with limited compliance.

## Figures and Tables

**Table 1:** Demographic distribution and baseline pulmonary function characteristics of the SAD and non-SAD groups.

	SAD	Non-SAD	*p*-Value
N	1061	1449	
Age (years)	50.42 ± 18.32	41.04 ± 17.49	<0.001
Male *n* (%)	516(48.63)	696(48.03)	0.797
Female *n* (%)	545(51.37)	753(51.97)	–
Height (cm)	163.21 ± 11.38	164.94 ± 12.38	<0.001
Weight (kg)	66.94 ± 16.00	68.80 ± 19.30	0.0465
FEF_25–75_ (%pred)	0.43 ± 0.17	0.88 ± 0.18	<0.001
FEF_50_ (%pred)	0.49 ± 0.20	0.94 ± 0.19	<0.001
FEF_75_ (%pred)	0.35 ± 0.14	0.77 ± 0.25	<0.001
FVC (%pred)	0.98 ± 0.17	1.06 ± 0.14	<0.001
FEV1 (%pred)	0.80 ± 0.15	0.85 ± 0.19	<0.001
FEV1/FVC (%)	68.80 ± 11.94	83.12 ± 5.07	<0.001
R5 (kPa/L/s)	0.47 ± 0.20	0.37 ± 0.14	<0.001
R20 (kPa/L/s)	0.32 ± 0.09	0.29 ± 0.08	<0.001
R35 (kPa/L/s)	0.39 ± 0.11	0.36 ± 0.10	<0.001
X5 (kPa/L/s)	−0.16 ± 0.12	−0.10 ± 0.05	<0.001
Fres (Hz)	17.56 ± 6.25	13.63 ± 3.96	<0.001
Z5 (kPa/L/s)	0.50 ± 0.22	0.38 ± 0.14	<0.001

**Table 2:** Five-fold cross-validation performance of the proposed model on the main task. Fold1–Fold5 denote the results on each held-out test fold.

	Accuracy	Specificity	Precision	Sensitivity	F1 Score
Fold1	0.8187	0.8379	0.8467	0.7924	0.7869
Fold2	0.8147	0.8586	0.8272	0.7547	0.7748
Fold3	0.8088	0.8793	0.8070	0.7123	0.7588
Fold4	0.8147	0.8724	0.8188	0.7358	0.7704
Fold5	0.8127	0.9139	0.7934	0.6745	0.7526
Average	0.8139	0.8724	0.8186	0.7340	0.7687

**Table 3:** Performance comparison with baseline methods for SAD identification.

	Accuracy	Sensitivity	Precision	Specificity	F1-Score
XGBoost	0.6926	0.4834	0.6939	0.8448	0.5698
ResNet10	0.7151	0.5000	0.7413	0.8724	0.5971
ResNet18	0.7291	0.5000	0.7794	0.8966	0.6092
ResNet34	0.7111	0.5472	0.6914	0.8138	0.6253
GoogLeNet	0.7331	0.5472	0.7532	0.8690	0.6339
Transformer	0.7510	0.6085	0.7544	0.8552	0.6736
Ours	0.8139	0.7340	0.8186	0.8724	0.7687

**Table 4:** Ablation study on the contributions of different input branches.

Time-Series Data	TFRIM	Demographics	Accuracy	Sensitivity	Precision	F1
✓			0.7430	0.6415	0.7196	0.6783
	✓		0.7490	0.6651	0.7194	0.6912
		✓	0.5816	0.6509	0.6765	0.6634
✓	✓		0.7749	0.6321	0.7929	0.7034
✓		✓	0.7629	0.6415	0.7598	0.6957
	✓	✓	0.7689	0.6179	0.7892	0.6931
✓	✓	✓	0.8139	0.7340	0.8186	0.7687

**Table 5:** Performance comparison of demographic feature fusion mechanisms.

Fusion Strategy	Accuracy	Sensitivity	Precision	F1
Concatenation Fusion	0.7849	0.6368	0.8133	0.7143
Gating Fusion	0.7809	0.6792	0.7742	0.7236
Additive Bias Calibration Fusion	0.7968	0.7476	0.7621	0.7548
DAFM Fusion	0.8139	0.7340	0.8186	0.7687

**Table 6:** Performance of individual subtasks and the final voting-based SAD identification.

	Accuracy	Sensitivity	Precision	Specificity	F1-Score
FEF_25–75_	0.8139	0.7366	0.8034	0.8693	0.7673
FEF_50_	0.8215	0.6574	0.7944	0.9079	0.7170
FEF_75_	0.7769	0.8446	0.8132	0.6572	0.8278
SAD	0.8139	0.7340	0.8186	0.8724	0.7687

## Data Availability

Anonymized individual patient data analyzed during this study are available from the corresponding author on reasonable request. Approval of such requests depends on the nature of the request, merit of the research proposed, availability of the data, and intended use of the data. Data requests should be sent to zfang@mail.ie.ac.cn.

## Abbreviations

The following abbreviations are used in this manuscript:

SAD	small airway dysfunction
IOS	impulse oscillometry
MBNW	multiple breath nitrogen washout
HRCT	high resolution computed tomography
TFRIM	time–frequency respiratory impedance map
DFT	discrete Fourier transform
FVC	forced vital capacity
FEF_25–75_	the mean forced expiratory flow between 25% and 75% of FVC
FEF_50_	the forced expiratory flow when 50% of FVC has been exhaled
FEF_75_	the forced expiratory flow when 75% of FVC has been exhaled
CNN	convolutional neural network
MLP	multi-layer perceptron
DAFM	demographics-driven adaptive feature modulation
STFT	short-time Fourier transform
SNR	signal-to-noise ratio
CBAM	convolutional block attention module
